# Breast Cancer with Brain Metastasis: Molecular Insights and Clinical Management

**DOI:** 10.3390/genes14061160

**Published:** 2023-05-26

**Authors:** Mariia Ivanova, Francesca Maria Porta, Federica Giugliano, Chiara Frascarelli, Elham Sajjadi, Konstantinos Venetis, Giulia Cursano, Giovanni Mazzarol, Elena Guerini-Rocco, Giuseppe Curigliano, Carmen Criscitiello, Nicola Fusco

**Affiliations:** 1 Division of Pathology, IEO, European Institute of Oncology IRCCS, 20141 Milan, Italy; mariia.ivanova@ieo.it (M.I.); francescamaria.porta@unimi.it (F.M.P.); chiara.frascarelli@ieo.it (C.F.); elham.sajjadi@ieo.it (E.S.); konstantinos.venetis@ieo.it (K.V.); giulia.cursano@ieo.it (G.C.); giovanni.mazzarol@ieo.it (G.M.); elena.guerinirocco@ieo.it (E.G.-R.); 2 School of Pathology, University of Milan, 20122 Milan, Italy; 3 Department of Oncology and Hemato-Oncology, University of Milan, 20122 Milan, Italy; federica.giugliano@ieo.it (F.G.); giuseppe.curigliano@ieo.it (G.C.); 4 Division of Early Drug Development for Innovative Therapies, IEO, European Institute of Oncology IRCCS, 20141 Milan, Italy

**Keywords:** breast cancer, metastatic breast cancer, brain metastasis, molecular profiling, biomarkers

## Abstract

Breast cancer is the most frequently diagnosed malignancy worldwide and the leading cause of cancer-related death among women. Brain metastases are a primary contributor to mortality, as they often go undetected until late stages due to their dormant nature. Moreover, the clinical management of brain metastases is complicated by the relevant issue of blood-brain barrier penetration. The molecular pathways involved in the formation, progression, and colonization of primary breast tumors and subsequent brain metastases are diverse, posing significant hurdles due to the heterogeneous nature of breast cancer subtypes. Despite advancements in primary breast cancer treatments, the prognosis for patients with brain metastases remains poor. In this review, we aim to highlight the biological mechanisms of breast cancer brain metastases by evaluating multi-step genetic pathways and to discuss currently available and emerging treatment strategies to propose a prospective overview of the management of this complex disease.

## 1. Introduction

Breast cancer (BC) is the most frequently diagnosed malignancy worldwide and the leading cause of cancer-related death in the female population [1]. Significant improvements in diagnosis and treatment strategies have substantially enhanced the management of these patients [2]. However, when the tumors have progressed to an advanced stage, the outcome remains poor, with a 5-year relative survival rate of 31% for de-novo metastatic BC (MBC) [3,4]. The most common anatomical sites of BC metastatic deposits include bone, liver, lung, and brain, with the latter being one of the major causes of mortality [5,6,7]. Of note, brain metastases (BMs) are more frequent in patients with HER2+ or triple-negative BC (TNBC) [3,6,8].

Patients with intracranial disease require multidisciplinary therapeutic approaches, including surgery, radiation, and pharmacological therapies [9,10]. Among these, medical treatments are particularly challenging due to the presence of the blood–brain barrier (BBB), which may limit the successful access to the central nervous system (CNS) of bioactive compounds [11]. Due to the complex crosstalk between the tumor and neural microenvironment, it is paramount to dissect the molecular mechanisms of BC with BMs. This would allow for the optimization of the existing systemic treatments and the critical development of novel and more effective strategies.

In this review article, we provide an overview of the biological mechanisms underlying the development of BMs in patients with BC along with the currently available and emerging medical pharmacological treatment options based on known molecular mechanisms. 

## 2. Biological Mechanisms of Brain Metastasis

Systemic metastases are the result of a multi-stage process that involves the detachment of the neoplastic cells from the primary tumor mass, and their migration through the local mesenchyme into the blood and lymphatic vessels [12]. This process requires reciprocal interactions between tumor cells and host tissues that involve epithelial-to-mesenchymal transition (EMT), changes in adhesion, proteolysis, invasion, and angiogenesis [13,14]. 

In BC, these events are linked to the alteration of the Wnt signaling pathway and inactivation of cadherin 1 (CDH1) [15,16,17]. Once neoplastic cells reach the peritumoral microenvironment, their survival relies on the so-called intravasation, which is the ability to invade the systemic circulation and reach distant anatomical sites [18]. The brain is a richly vascularized organ and is therefore exposed to a high amount of circulating tumor cells, provided that they can cross the BBB [19]. This barrier is composed of a layer of specialized endothelial cells expressing a specific subset of membrane transporters and pumps that allow tight regulation of the brain microenvironment and are impermeable to most foreign agents [20]. Astrocytes further contribute to the regulation of the BBB by forming a second basement membrane around brain capillaries through their cytoplasmic foot processes [21]. Still, some BC cells can cross the BBB and invade the brain parenchyma. 

**Figure 1:**
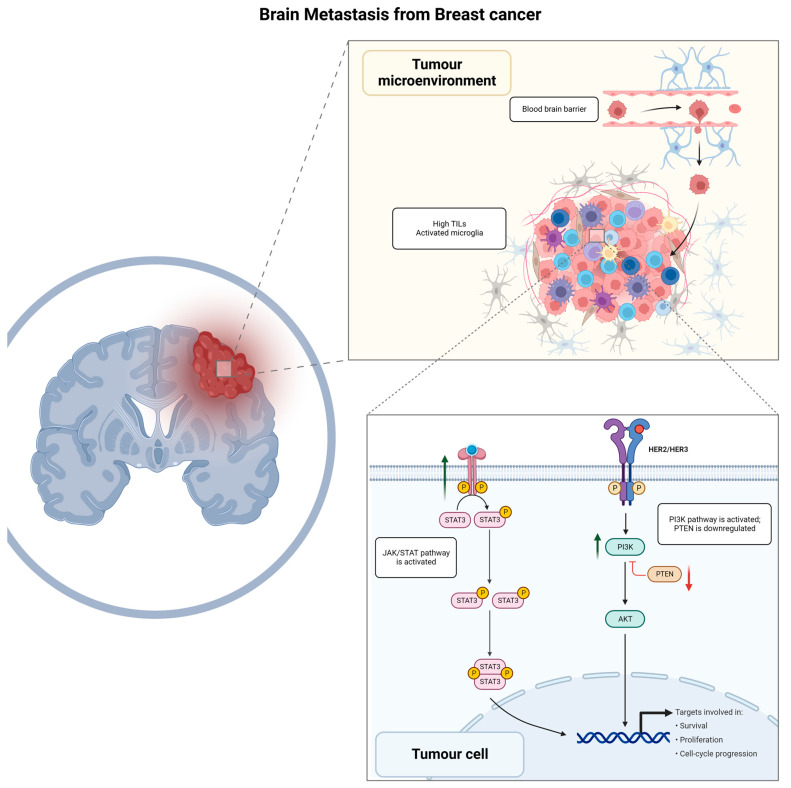
Graphical summary of biological mechanisms involved in brain metastasis development for breast cancer patients. TILs, tumor infiltrating lymphocytes; JAK/STAT, Janus kinase signal transducer and activator of transcription; HER2/HER3, human epidermal growth factor receptor 2/human epidermal growth factor receptor 3; PI3K/AKT/PTEN, phosphoinositide 3—kinase-protein kinase B (Akt)—phosphatase and tensin homolog.

Colonization of the brain by BC starts with the adhesion of the circulating tumor cells to the capillary endothelium [22,23]. Upregulation of the membrane glycosyl-transferase ST6GALNAC5 has been demonstrated to play an important role in this process [24]. Its expression is normally restricted to the brain, but BC neoplastic cells that acquire the ability to synthesize it have an increased ability to cross the BBB and invade the brain parenchyma [25]. In addition, β4 integrin signaling promotes tumor endothelial adhesion and extravasation by enhancing vascular endothelial growth factor (VEGF) expression, activated by hypoxia, which promotes vascular remodeling and increased permeability [26,27,28]. After the BBB has been breached, reactive astrocytes activated by contact with cancer cells initiate an anti-tumoral response by secreting plasminogen activators [29]. In the early phases of the anti-tumoral response, this promotes the activation of plasmin, which in turn is responsible for the elimination of neoplastic cells [30]. However, some tumor cells can escape by producing anti-plasminogen activator serpins [31]. In the later phases of metastasis growth, reactive astrocytes have a tumor-promoting effect through the creation of a microenvironment that favors metastasis progression [32]. The summary of BC BM biological mechanisms is represented in Figure 1.

### 2.1. JAK/STAT Signaling Pathway

Reactive astrocytes following BM are characterized by activation of the JAK/STAT signaling pathway that promotes tissue repair and scar formation following brain injury [33]. Astrocytes expressing pSTAT3 increase the recruitment of CD74+ microglia-macrophages, which in turn induces the activation of the macrophage migration inhibitory factor (MIF) [34,35]. The activation of the CD74-MIF axis has an immunosuppressive effect on peritumoral microglia macrophages, creating a metastasis-favorable microenvironment [36]. In addition, STAT3+ astrocytes have an immune suppressive effect by inducing the expression of programmed cell death–1 (PD-1) and programmed cell death–1 ligand 1 (PD-L1) [37,38,39,40,41]. Furthermore, BC BM has been observed to be highly immunogenic, with high levels of tumor-infiltrating lymphocytes (TILs) frequently reported within the tumor and the surrounding stroma [42,43]. 

### 2.2. Immune Checkpoints Mechanisms

A study of 233 patients with solid tumors of various origins and concurrent BM has demonstrated PD-L1 expression in 23.6% of BMs, wherein 18% (19 cases) showed PD-L1 expression in both the primary tumor and the BM (22C3 anti-PD-L1 antibody, Dako Agilent) [44]. Interestingly, authors have also shown that IHC evaluation of CD8 (a co-receptor for the T-cell receptor) resulted in its expression being associated with higher PD-L1 expression, both in the primary tumor and BM, which confirms the ongoing lymphocytic reaction in the BM microenvironment [44]. Significant correlations were identified between the infiltration of CD8-positive lymphocytes in primary tumors and the BM characteristics, with a higher incidence of multiple BMs observed in cases with lower levels of CD8 infiltration in the primary tumors. [44,45]. Griguolo et al. have also confirmed that TNBC BMs showed a significantly higher percentage of intra-tumoral CD8+ cells and a higher density of CD163+ M2-polarized microglia/macrophages within the HER2-negative BC BMs, associating the latter with a worse prognosis, but identifying another potential therapeutic target to be explored [46]. The authors emphasize that, paradoxically, in both TNBC BMs and HR+/HER2− BC BMs, the interaction between CD163+ microglia/macrophages and T lymphocytes was associated with a better outcome [46]. A study by Noh et al. exploring the evolution of the tumor microenvironment in BC BM found that a lower CD8+ T cell count, low CD86+ M1 macrophage count, and high M2/M1 macrophage ratio in the BC BM compared to the primary tumor were related to unfavorable clinical outcomes [47]. Furthermore, Giannoudis et al. have conducted an analysis of 55 samples consisting of 26 paired primary BCs and their BMs, assessing TILs and mRNA expression [48]. Authors have demonstrated a significant reduction of TILs in BC BMs in comparison to primary BCs, with an 11.5% high-TILs count in primary tumors (>40% stromal TILs) versus only 3.8% in BC BMs [48]. A total of 112 immune-related gene levels were found to decrease in BC BMs compared to primary BCs, including PD-L1 and cytotoxic T-lymphocyte antigen 4 (CTLA-4) (false discovery rate < 0.01, log2 fold-change > 1.5), which are involved in cytokine–chemokine signaling, immune cells migration, matrix remodeling, and metastasis [48]. CTLA-4 is one of the fundamental immunosuppressive cytokines, mainly activated on T-cells, which suppresses the immune response, and potentially prevents cancer cells from being attacked by the immune system. CTLA-4 genetic variants have been shown to play a role in BC progression, presenting a prognostic value [49] and making CTLA-4 a potentially attractive target for BC immunotherapeutic approach development, wherein the evaluation of its mutations may become markers for genomics-based precision medicine and effective BC treatment [50]. The depletion of T-cell response could be driven by *ARG2* (Arginase 2) upregulation, which was found in BC BMs and confirmed immunohistochemically (ARG2 protein expression was associated with worse breast–brain metastasis-free survival (*p* = 0.027) and OS (*p* = 0.019)), so ARG2 could be another potential marker of BC distant metastasis and a therapeutic target in BC BM, as proposed by authors [50]. The model of mouse mammary carcinoma by Sham et al. has provided an analysis of EMT cell lines, wherein tumor cells acquired resistance to radiotherapy, changing their phenotype and causing them to acquire higher migratory and survival rates, which in turn represents a higher metastatic potential [14]. The authors performed a next-generation sequencing (NGS) to explore underlying genes responsible for EMT cell culture and found upregulation of *PDL-1, AXL, GAS6,* and *APCDD1*, which are believed to contribute to radioresistance acquisition through the JAK/STAT/PI3K pathway [14]. Based on this hypothesis, the authors determined the levels of PD-1 and CTLA-4 proteins expression, as they are known to be associated with the JAK/STAT/PI3K pathway [51]. Indeed, these proteins were confirmed to be overexpressed in the EMT cell line by Western blot [14]. The immune microenvironment has a profound influence on the outcome of patients with BMS from BC, with a significantly poorer prognosis in tumors with increased PD1/PDL1 expression [46]. However, these patients could benefit from targeted immune therapy [52,53]. 

### 2.3. PI3K-Akt Signaling Pathway

The activation of the phosphoinositide 3-kinase (PI3K)-protein kinase B (Akt) signaling pathway has been observed in a large proportion of BCs with BM [54,55]. Activation of this pathway leads to a more aggressive phenotype of neoplastic cells, with increased survivability, proliferation, and angiogenetic potential [56,57]. Patients with BMs harboring activation of the PI3K-Akt signaling pathway have a worse outcome compared to patients with the same-stage disease and lower PI3K-Akt activity [54]. In addition, it contributes to the modulation of the peritumoral immune microenvironment through the activation of immunosuppressive regulators such as PD-1 and CTLA-4 [39]. Interestingly, a functional link has been reported between the PI3K and STAT3, where a synergistic interaction of the two pathways has been observed in murine neoplastic cells [58]. This crosstalk is mediated by the cytoplasmic tyrosine-protein kinase BMX, a member of the TEC kinase family known to be a STAT3 phosphorylator [59,60]. TEC kinases, including BMX, are in turn activated by PI3K, with a positive effect on STAT3 activation [61]. This inter-dependency is further demonstrated by the fact that PI3K inhibition significantly reduces STAT3 phosphorylation and activation [62].

Another important component activating the PI3K-AKT pathway is HER2-HER3 dimerization [63], which plays a central role in the biology of BC with BM [54,57,64,65]. Patients with HER2+ disease are at increased risk of developing BMs [3,8,66], with HER2 overexpression often preserved at the metastatic site, and associated with the overexpression of HER3 [63], a coreceptor that forms heterodimers with HER2, thus playing a role in the neoplastic transformation of HER2-enriched BCs [67]. This provides a further therapeutic perspective on patients with BMs from BC, as HER3 inhibitors are available and have been proven to be effective in increasing the sensitivity to PI3K inhibitors [68].

### 2.4. PTEN

Another frequent occurrence in BC BM is the loss of phosphatase and tensin homolog (PTEN) [69]. This event has been observed to be more frequent in BM compared to dissemination to other organs, suggesting a role of the local microenvironment in inducing this alteration [70]. This mechanism has indeed been demonstrated to depend on epigenetic regulation through micro-RNAs (miRNAs) secretion by astrocytes [71]. In addition, PTEN loss also induces the secretion of cytokine–chemokine ligand 2 (CCL2), which is responsible for the creation of a pro-inflammatory microenvironment [72], and recruitment of ionized calcium-binding adapter molecule 1 (Iba1)+ myeloid cells that reduce apoptosis and promote growth in neoplastic cells [71].

## 3. Patients at High Risk

Numerous achievements in medical treatment have improved overall disease control in this population. Nevertheless, in clinical practice, the rate of intracranial progression, while extracranial disease appears to be under control, is growing [73]. BMs are more frequently observed in HER2+ and TNBC subtypes [73,74]. In addition to the abovementioned risk of a certain molecular subtype, a young age at the initial diagnosis, larger tumor size, higher histologic grade, higher tumor stage, high proliferation Ki67 index, presence of lung metastases, and *BRCA1* phenotype increase the risk of BM in BC patients [73,74,75].

TNBC-derived BMs have been reported to arise earlier compared to those of other molecular subtypes, with a significantly shorter survival, a median overall survival (OS) of roughly six months and the worst BC-specific survival and OS [74].

A survival analysis of 83 HER2-positive metastatic BC patients found that hormone-negative tumors and larger BMs were related to a poorer prognosis, as well as the use of single-agent chemotherapy with trastuzumab, and the use of only one type of anti-HER2 agent after trastuzumab resulted in a poor prognosis [76]. These data are supported by a previous study of 228 HER2-positive BC patients, of whom 51 had BMs and a poor prognosis due to the lack of effective treatments for BM [73]. 

Hormone status, as an unequivocal pillar of BC patients’ assessment, has given some insights into BM development risk. Estrogen supplementation to premenopausal levels can enhance the risk of BM in TNBC patients [74]. Estrogen-treated astrocytes have been demonstrated to activate epidermal growth factor receptor (EGFR) in premenopausal women via epidermal growth factor receptor EGFR ligand overexpression, and play an important role in promoting invasion and BM colonization. The paracrine effect of estrogen may promote metastases, whereas estrogen deprivation has been shown to significantly lower brain metastatic load in triple-negative EGFR + tumors [77].

Progesterone, having an impact on mammary epithelial cell proliferation and differentiation, is also a risk factor for BC. In TNBC, progesterone has been demonstrated to reduce TNBC cell proliferation, progression, and BM via the membrane progesterone receptor α, suggesting the progesterone-mPRα pathway as a target [78].

Epigenetic changes have also been considered significant in the evolution of BC. A meta-analysis of epigenetic genes identified the enhancer of zeste homolog 2 (*EZH2*) as a key driver of TNBC. TNBC cells with increased *EZH2* expression were more likely to metastasize, whereas inhibition of *EZH2* activity reduced tumor spread and metastasis [79]. Highly expressed TNBC-specific transcription factor Engrailed 1 (*EN1*) is linked to the regulation of genes related to neurogenesis. Its high expression has been found to correlate with short overall survival and increased risk of developing brain metastases in patients with TNBC, which describes *EN1* as a prognostic marker and a potential therapeutic target [80].

Resent research has focused on novel predictive biomarkers identification, to improve high-risk patients’ stratification. The investigation of insulin resistance markers and fasting triglyceride–glucose (TyG) indices in HER2-positive BC patients with BMs suggests that the TyG index could be used as a predictive biomarker at the time of diagnosis for the risk of time to BM, with a need for prospective studies confirming these data [81].

## 4. Targeting Therapies for Breast Cancer Patients with Brain Metastasis

According to the latest ASCO-SNO-ASTRO guidelines, surgery with subsequent radiotherapy on the operated field may be offered for patients with one brain mass without primary cancer diagnosis or for large tumors with mass effect. Local treatment with radiotherapy is reasonable for symptomatic BMs, regardless of the systemic therapy administration [82]. There is not one recommended sequence for patients receiving both local treatments and systemic therapy [83]. Medical treatments for BC are mainly based on hormone receptor and HER2 status, while some novel target therapies are available depending on the tumor molecular profile [7,82,84]. Pharmacological approaches for patients harboring encephalic disease do not differ from patients who do not, except for HER2-positive BC, for which guidelines providing a specific treatment algorithm have been provided [85]. 

### 4.1. HR+/HER2−—Breast Cancer

Local treatments (surgery and radiation therapy) remain the gold standard for BMs from HR-positive/HER2-negative (HR+/HER2−) BC [82,83]. Existing practice guidelines for MBC treatment recommend sequential endocrine/targeted therapy until the exhaustion of available agents, before systemic cytotoxic chemotherapy administration [83,86,87]. Although BMs in HR+/HER2− BC show a lower incidence compared to HER2+ and TNBC subtypes, endocrine therapy has been found to be beneficial for both CNS and systemic management [83,88]. Indeed, tamoxifen and its metabolites have been reported to achieve effective concentrations in the CNS [89]. First-line treatment for this population is based on endocrine therapy plus cyclin-dependent kinase 4/6 (CDK4/6) inhibitors, namely palbociclib, ribociclib, or abemaciclib [90]. These three agents are capable of crossing the BBB, but there is a lack of clinical data to inform a CNS-specific response rate. Indeed, no data on CNS outcomes are available for ribociclib and palbociclib [83]. Regarding abemaciclib, a single-arm phase II clinical trial did not meet its primary endpoint of intracranial overall response rate in HR-positive BC with BMs, although the drug achieved an excellent concentration in cerebrospinal fluid. It is worth noting that palbociclib has been shown to be effective in BMs of different tumor types, including BC, and to harbor a CDK4/6 alteration [91]. Companion endocrine therapy for CDK4/6 inhibitors could be represented by fulvestrant or letrozole. Elacestrant, an oral selective estrogen receptor degrader approved by the FDA, could be an option for patients harboring an *ESR1* mutation after CDK4/6 exposure [92]. However, no data on CNS efficacy are available for this drug at the time of writing.

Furthermore, preclinical data have shown that the serine/threonine protein kinase AKT (protein kinase B) inhibitor capivasertib is active in combination with fulvestrant, providing the basis for future studies [93]. Given the promising results of the phase II FAKTION trial (NCT01992952) [94,95], capivasertib was further investigated in the phase III CAPItello-291 trial (NCT04305496), with results recently reported at the San Antonio Breast Cancer Symposium 2022 [96]. Although patients with BMs were included, results in this subgroup are pending. 

Another crucial pathway in BC is the PI3K/Akt/mammalian target of rapamycin (mTOR) signaling, wherein activating *PIK3CA* mutations, occurring in about 40% of hormone receptor HR+/HER2− MBCs, are driver events for tumorigenesis and tumor progression [57]. This pathway has been successfully targeted by the α-selective PIK3CA inhibitor alpelisib [55], with a manageable safety profile (mainly diarrhea and hyperglycemia). The incidence of BMs is particularly high among patients with HR+/HER2− BC patients carrying a *PIK3CA* mutation [97], and one report has shown a gain of *PIK3CA* mutation in BM samples despite its absence in a primary BC [98]. Real-world evidence suggests that alpelisib may have CNS activity [99]. However, the low number of cases reported justifies the need for additional investigations to prospectively evaluate alpelisib efficacy in patients with BC BM [100].

### 4.2. Triple-Negative Breast Cancer

Among the three main BC subtypes, TNBC is characterized by a limited therapeutic armamentarium [101]. However, this scenario is rapidly expanding thanks to innovative options based on the characterization of individual tumor molecular profiles [102,103,104,105]. At the moment, the treatment strategy for most TNBCs relies on chemotherapy, immunotherapy (if PD-L1 is positive), and target therapy with poly (adenosine diphosphate–ribose) polymerase inhibitors (PARPis) based on germline *BRCA1/2* (*gBRCA1/2*) status. [85,106,107,108]. In patients with MBC harboring a *gBRCA1/2* pathogenetic variant, the two PARPis, talazoparib and olaparib, showed a benefit in survival outcomes [109]. Regarding BMs, a higher frequency of BMs is reported in this population. In the EMBRACA trial (NCT01945775), talazoparib resulted in significantly longer progression-free survival than standard-of-care chemotherapy; this was also found in the subgroup of patients with CNS metastases [110]. However, the final overall survival analysis was not statistically significant [111]. It is worth noting that the enrolled population comprised both TNBC and HR+ BC; no specific analysis for BMs in each subgroup is available, given the limited number of patients with BMs enrolled in the study (about 15%). The same concern could be raised for the OlympiAD trial, although in this study no specific subgroup analysis for BMs is available [112]. In these studies, PARPis have shown mainly hematological and gastrointestinal toxicity. 

On the other hand, immunotherapy has demonstrated potential intracranial efficacy among patients with MBC [113,114]. Indeed, recent studies demonstrated that TNBCs exhibit higher PD-L1 expression than other BC subtypes, which is associated with remarkable genomic instability and higher immune infiltration [115]. The role of the immune microenvironment and PD-L1 expression in the brain and metastatic brain tumors is poorly understood [46,48]. Chehade and colleagues have studied 59 immunotherapy-naïve BC patients with BMs in a single-center retrospective cohort study, wherein 15.3% had PD-L1+ BM, with the highest proportion (25%) among those with TNBC (SP142 antibody, Ventana) [52]. In this study, the concordance in PD-L1 expression between primary BC and BM in TNBC specifically could not be studied due to small sample sizes, but authors emphasize that PD-L1 expression has previously been reported to be higher in the primary tumor compared to metastatic sites (63.7% vs. 42.2%, *p* < 0.0001), specifically in TNBC [52,116]. Hence, it was proposed that PD-L1 staining should be performed both on the primary and metastatic tumor to maximize the opportunity for ICI therapy administration [116]. 

Focusing on clinical data, the combination of chemotherapy and pembrolizumab improved overall survival with manageable safety in patients with PD-L1+ (CPS ≥ 10) advanced TNBC in the phase III KEYNOTE-355b study [117]. In this study, only a few patients with BMs were enrolled; thus, it is not possible to derive any conclusion [118,119]. The IMPASSION-130 trial (NCT03483012) has demonstrated a benefit of atezolizumab in combination with nab-paclitaxel for TNBC treatment; however, no progression-free survival (PFS) benefit for BM patients has been observed, although this population had a relatively small representation (6.3%) [120].

### 4.3. HER2+ Breast Cancer

In HER2+ BC subtypes, one of the primary therapeutic targets is human epidermal growth factor receptor 2 (HER2), overexpressed in about 20% of cases [121]. Interestingly, some studies demonstrated that several primary BCs, originally negative for HER2, developed HER2 positivity after metastasizing to the brain [122]. HER2− and EGFR-targeted therapies are being evaluated and used in the clinic and/or evaluated in clinical trials for BC patients with BM treatment [123]. The results of the phase III trial CLEOPATRA (NCT00567190) in HER2+ MBC patients have demonstrated significant improvements in progression-free and overall survival with the administration of pertuzumab, trastuzumab, and docetaxel over placebo, trastuzumab, and docetaxel [124]. Swain et al. have carried out exploratory analyses of the incidence and time to development of CNS metastases in the same patients’ cohort, revealing that pertuzumab, trastuzumab, and docetaxel delay the onset of CNS disease compared with placebo, trastuzumab, and docetaxel [125], maintaining this effect for more than 8 years of median follow-up [126]. Another phase III study, TH3RESA (NCT01419197), compared the effect of trastuzumab emtansine (T-DM1) administration with other treatments of the physician’s choice in patients with HER2+ metastatic BC, showing that T-DM1 reduces the risk of disease progression in patients with BMs at baseline [127]. This effect was confirmed by a more recent KAMILLA study (NCT01702571), wherein T-DM1 demonstrated CNS-specific benefit in patients with HER2+ BC with BMs [128], supporting the manageable safety profile and use of T-DM1 in advanced BC [129].

The randomized, double-blind, placebo-controlled HER2CLIMB clinical trial (NCT02614794) compared a small-molecule highly selective for HER2 oral tyrosine kinase inhibitor (TKI), tucatinib, versus placebo in combination with trastuzumab and capecitabine in patients with HER2-positive metastatic BC previously treated with trastuzumab, pertuzumab, and T-DM1, which resulted in tucatinib’s approval by the FDA in 2020, also applicable for BC patients with BMs [130]. The objective response rate was doubled by the addition of tucatinib to trastuzumab and capecitabine in HER2+ BC patients with BMs, reducing the risk of intracranial progression or death by two-thirds, and reducing the risk of death by nearly half [131]. Peculiar adverse events of the combination with tucatinib included diarrhea, palmar–plantar erythrodysesthesia syndrome, nausea, fatigue, vomiting and elevated aminotransferase levels.

An irreversible oral pan-ERBB inhibitor targeting HER1, HER2, and HER4, pyrotinib, has been demonstrated to be another promising agent for the treatment of HER2+ metastatic BC; however, there are no data on cohort stratification of the type of metastasis available [9]. 

The novel biparatopic anti-HER2 antibody–tubulin conjugate (bHER2-ATC) has been investigated in a preclinical murine BC model with BMs, showing the increased uptake of the agent by endocytosis and suggesting a possible effective mechanism of brain penetration [132].

Trastuzumab deruxtecan (T-DXd) has shown durable antitumor activity in pretreated patients with HER2-positive MBC, and its efficacy in patients with leptomeningeal disease and active BM (BMs) is under evaluation. In the single-arm phase II TUXEDO-1 trial, T-DXd showed a high intracranial response rate in patients with HER2-positive BC and newly diagnosed untreated BMs or BMs progressing after previous local therapy. Two patients (13.3%) had a complete intracranial response, nine (60%) had a partial intracranial response and three (20%) had stable disease, with a best overall intracranial response rate of 73.3% (95%CI 48.1–89.1%), maintained over treatment duration. The DEBBRAH trial aimed to assess T-DXd in patients with HER2-positive or HER2-low MBC and BMs. This five-cohort, phase II study (NCT04420598) enrolled patients with pretreated HER2-positive or HER2-low MBC with stable, untreated, or progressing BMs and/or leptomeningeal carcinomatosis. Authors reported findings from HER2-positive MBC patients with non-progressing BMs after local therapy (*n* = 8; cohort 1), asymptomatic untreated BMs (*n* = 4; cohort 2), or progressing BMs after local therapy (*n* = 9; cohort 3). Among patients with measurable intracranial or extracranial lesions at baseline, the ORR was 66.7% (12 out of 18 patients; 95%CI, 41.0–86.7); 80.0% (95%CI, 28.4–99.5) in cohort 1, 50.0% (95%CI, 6.7–93.2) in cohort 2, and 66.7% (95%CI, 29.9–92.5) in cohort 3. All responders had partial responses. T-DXd showed intracranial activity with a good safety profile and maintained the quality of life in these patients [133,134]. Most treatment-emergent adverse events were gastrointestinal and hematological in nature, but clinicians should be aware of the incidence of drug-related interstitial lung disease in the management of these patients. Currently, ASCO-SNO-ASTRO Guidelines recommend that patients with HER2+ BC with asymptomatic BMs, who have progressed on previous trastuzumab, pertuzumab, and/or trastuzumab emtansine-based therapy, may be given the combination of tucatinib, trastuzumab, and capecitabine. The evidence suggests a possible benefit of postponing local therapy using these agents until evidence of intracranial progression is seen [10,14].

## 5. Future Perspectives

Although individuals with BC BMs are mostly disproportionately underrepresented in pertinent clinical trials, the research target therapies’ spectrum amplification is ongoing. One of the remaining challenges in metastatic BC treatment is its lower efficacy in a BM setting due to the presence of the BBB, since most antitumor drugs do not cross the blood–brain barrier [52,53]. The summary of actual BC brain metastasis targeting agents and their corresponding clinical trials is given in Supplementary Table S1; in Supplementary Table S2, only trials that were complete at the time of writing were reported.

Further investigations in the genomic landscape of BC are widening with the increasing availability of next-generation sequencing. This has led to the recognition of BC, and TNBC in particular, as a heterogeneous disease both clinically and molecularly, with different prognostic and therapeutic implications, which also complicates the prediction of the risk of metastasis [6,85]. Continuous efforts are being made to standardize present molecular assays and develop more reliable and reproducible testing for hormone receptor and HER2 gene expression so that the categorization of molecular subtypes can be more effective [98]. Molecular profiling is an essential prognosis determinant in the field of solid tumors in terms of understanding the nature of the disease and response to treatment [135]. One of the biggest analyses of the genomic landscape in a retrospective cohort study of 733 BC BM and 10,772 primary BC specimens revealed that BMs were enriched in genomic alterations of *TP53* (72.0%, 528/733), *ERBB2* (25.6%, 188/733), *RAD21* (14.1%, 103/733), *NF1* (9.0%, 66/733), *BRCA1* (7.8%, 57/733), and *ESR1* (6.3%,46/733) (*p* < 0.05) [98]. The authors further suggest that both surgical specimens and cerebrospinal fluid are suitable and may be used for comprehensive genomic profiling in the research of novel therapeutic strategies [98].

Novel biomarkers research refers to the targeting of human trophoblastic cell surface antigen 2 (TROP-2), a transmembrane calcium signal transducer expressed on the membrane surface of epithelial cells in multiple tumors [136]. The monoclonal antibody sacituzumab govitecan, targeting TROP-2, is currently under investigation in a phase II clinical trial by the Southwest Oncology Group (NCT04647916) in patients with HER2-negative BC with BMs. The primary objective is the assessment of the intracranial objective response rate; the secondary objectives are OS, PFS, safety, and the tolerability of the drug. Additionally, the trial aims to evaluate patients by stratifying them by hormone-receptor subtype [137].

Datopotamab deruxtecan (Dato-DXd) is another potential drug within the group of antibody–drug conjugates (ADCs), targeting TROP2 [138]. Its effect has been specifically studied in the TNBC and HR+ HER2-negative forms of advanced BC, demonstrating a substantial clinical benefit; however, no data on BMs specifically are present [138,139].

Another promising developing treatment option is nanotherapy. This approach consists of intravenous drug delivery, conjugating nanoparticles to therapeutic agents, and targeting overexpressed antigens or receptors [20]. The nanocarriers possess unique advantages, having a high drug-loading capacity and the ability to protect the enclosed agent from rapid clearance, forming a solid concentration gradient that enhances vascular permeability [9]. This therapy has been effective in brain cancer; however, the evidence on BC BMs is scarce and further investigations are warranted [9]. Screening is a critical issue in patients with early BC, wherein specifically the screening for asymptomatic BM is currently unjustified; hence, there is an urgent need for appropriate guidelines establishment [140].

Moreover, the multidisciplinary management of BMs should evolve according to the new acquisitions in terms of new drugs. Clinicians are rethinking the role of radiotherapy, investigating new possible ways to imbricate radiation and systemic treatment, for example postponing radiation if a new treatment with a high intracranial efficacy should be started (for example, tucatinib or T-DXd). However, evidence in this field is lacking, and data from real-world studies are awaited. 

## 6. Concluding Remarks

Identification of novel targetable biomarkers that might help establish treatment targets or prognosis indicators is ongoing, suggesting that the terminology for BC may also be revised in favor of guided therapy assistance and further characterization of the TNBC and other subtypes heterogeneity [105,141]. A significant clinical burden and an unmet need for more effective therapy in these high-risk BC patients are highlighted by the fact that TNBCs have a greater cumulative rate and more propensity for brain metastasis with the shortest DFS, compared to other BC subtypes [102]. Our knowledge of the incidence and CNS-specific effectiveness of systemic therapy in the high-risk TNBC patient population is limited since clinical studies frequently do not capture BMs and there is a pitfall present in asymptomatic BM screening rarity [8,140]. Research on TNBC, therefore, has to be enhanced and should increasingly focus on finding novel treatment targets. Due to the high incidence of BM among patients with metastatic HER2+ and TNBC, studies to determine the value of screening for BM should be undertaken in these subgroups [73,140]. Given the high prevalence of clinically relevant genomic alterations in patients with BC and BMs, comprehensive genomic profiling of the primary tumor tissue, as well as metastatic tissue in the brain or cerebrospinal fluid specimen, is suggested [98,142]. Epigenetic remodeling may be new emerging opportunity for these high-risk patients [74]. Prospectively, the development of combination therapy focusing on various cell types and adaptive immune response phases is foreseen [48]. The limitations of most available studies represent the low number of patients and the overall BC with BM cohort underrepresentation, resulting in a need for large multi-center studies to continue exploring the risk factors of BMs and the prognoses of these patients.

## Data Availability

No new data were created or analyzed in this study. Data sharing is not applicable to this article.

## Supplementary Materials

The following supporting information can be downloaded at: https://www.mdpi.com/article/10.3390/genes14061160/s1, Table S1: Breast cancer brain metastasis targeting agents and corresponding clinical trials.; Table S2: Breast cancer brain metastasis targeting agents and corresponding complete clinical trials.
