# Supplementary material for: Breast Cancer with Brain Metastasis: Molecular Insights and Clinical Management
Source: Genes (Basel). 2023 May 26;14(6):1160. doi: 10.3390/genes14061160 (PMC10298724; doi:10.3390/genes14061160)
Supplement: Supplementary file 1 [file genes-14-01160-s001.zip › genes-2404261-supplementary.pdf]

Intervention (drug)	Breast Cancer type	Phase	Status	Patients	Primary Outcome	Acronym	Trial Number
Pyrotinib (EGFRi) plus Vinorelbine (CT)	HER2+	II	U	30	ORR	Pyrotinib	NCT03933982
Capecitabine (CT) and Neratinib (TKI)	HER2-	II	R	22	OS, PFS	-	NCT04965064
Nivolumab (ICI)	BCBM	I	A	14	DLT	-	NCT03807765
Palbociclib (CDK4/6i), Trastuzumab (MA), Pyrotinib (TKI) and Fulvestrant (ERA)	ER+ HER2+	II	R	34	ORR	-	NCT04334330
Eribulin (CT)	HER2-	II	W	0	ORR	ERIBRAIN	NCT03637868
Utidelone (MTA)	HER2+, HER2-	II	R	100	ORR	-	NCT05357417
Trastuzumab deruxtecan (ADC)	HER2+	II	A	15	ReR	TUXEDO-1	NCT04752059
Tucatinib (TKI), Trastuzumab (MA), and Capecitabine (CT)	HER2+	I	NR	40	DLT	-	NCT05553522
Liposomal Doxorubicin	BCBM	II	T	2	ReR	-	NCT00465673
Neratanib (TKI)	HER2+	II	W	0	AEPIs	NeraBrain	NCT04856475
Trastuzumab (MA) and GDC-0084 (PI3Ki)	HER2+	II	R	47	ORR	-	NCT03765983
Trastuzumab (MA) and GRN1005 (PDC)	HER2+	II	C	85	ORR	GRABM-B	NCT01480583
Trastuzumab deruxtecan (ADC)	HER2+	III	R	500	ORR, PFS	DESTINY-B12	NCT04739761
BEEP regimen (bevacizumab (MA) followed by etoposide and cisplatin (CT)	BCBM	II	A	120	PFS	A-Plus	NCT02185352
ANG1005 (PDC)	BCBM	II	C	72	ORR	-	NCT02048059

Etirinotecan Pegol (polymer conjugate of TIi)	BCBM	III	C	178	OS	ATTAIN	NCT02915744
Temozolomide (CT)	BCBM	II	U	100	ORR	-	NCT00875355
Pembrolizumab (ICI)	BCBM with at least 2 brain metastases	I, II	R	41	ReR, OS	-	NCT03449238
Eribulin Mesylate (MTA)	BCBM	II	C	9	PFS	-	NCT02581839
ARX788 (ADC)	BCBM	II	R	32	CBR	-	NCT05018702
Trastuzumab Emtansine (ADC)	HER2+	I	C	36	optimal sequences of combined treatment	BIRTH	NCT02135159
QBS72S (CT)	Relapsed BCBM	II	R	40	ORR	-	NCT05305365
SHR-A1811 (ADC)	HER2+	II	NR	75	ORR	-	NCT05769010
Afatinib (TKI) and Trastuzumab Emtansine (ADC)	HER2+	II	R	130	DLT, ORR	HER2BAT	NCT04158947
Sorafenib (KI)	BCBM	I	C	21	MTD	-	NCT01724606
Pyrotinib (EGFRi),<br>Temozolomide (CT), SHR-1316 (ICI),<br>Bevacizumab (VEGFi)<br>Cisplatin/Carboplatin (CT)	HER2+, TNBC	II	U	59	ORR	-	NCT04303988
Olaparib (PARPi), Durvalumab (ICI)	BCBM	I, II	R	41	AEPIs, ORR	SOLARA	NCT04711824
Pyrotinib (EGFRi)	HER2+	I, II	A	39	S&T, LTCR	-	NCT04582968
Abemaciclib (CDK4/6i),<br>Elacestrant (ERA)	HR+ HER2-	I, II	R	44	AE, ORR	-	NCT04791384
Trastuzumab (MA)<br>Trastuzumab Emtansine (ADC)<br>Pertuzumab (MA)	HER2+	II	R	48	PFS	BRIGET	NCT05323955

Tucatinib (TKI)							
Pembrolizumab (ICI), Liposomal Irinotecan (TIi)	TNBC	II	NR	42	DCR	-	NCT05255666
Eribulin Mesylate (MTA)	BCBM	II	U	14	ORR		NCT03412955
Irinotecan Hydrochloride (TIi)	BCBM	II	R	63	ORR	Phenomenal	NCT03328884
Atezolizumab (MA)	TNBC	II	A	45	PFS	-	NCT03483012
Pyrotinib (EGFRi), Capecitabine (CT)	HER2+	II	A	78	ORR	-	NCT03691051
Lapatinib (TKI)	HER2+	I	C	35	MTD	-	NCT00470847
Capecitabine (CT), lapatinib (TKI)	HER2+	II	C	45	ORR	-	NCT00967031
Everolimus (KI), Vinorelbine (CT), Trastuzumab (MA)	HER2+	II	C	32	ReR	-	NCT01305941
Capecitabine (CT)	BCBM	II	T	24	ORR	XERAD	NCT00977379
Bevacizumab (MA)	HER2+	II	C	38	ORR	-	NCT01004172
HKI-272 (Neratinib, EGFRi)	HER2+	II	A	140	ORR		NCT01494662
Cabozantinib (TKI)	HER2+/ ER+/ TNBC	II	C	36	ORR	-	NCT02260531
INIPARIB (PARPi)	TNBC	II	C	44	TTP		NCT01173497
Efaproxiral (allosteric hemoglobin modifier)	BCBM	III	C	368	OS	ENRICH	NCT00083304
Trastuzumab Emtansine (ADC), metronomic Temozolomide (CT)	HER2+	I, II	A	12	MTD	-	NCT03190967
Eutidrone (CT), etoposide (CT), bevacizumab (MA)	BCBM	N/A	R	43	ORR	-	NCT05781633

Bevacizumab (MA), Docetaxel (CT), CPT-11 (CT), Paclitaxel (CT), Vinorelbine Tartrate (CT), Gemcitabine (CT)	BCBM	II	T	16	S&T	-	NCT00476827
ZK219477 (CT)	BCBM	II	T	15	ORR	-	NCT00496379
Trastuzumab Emtansine (ADC)	HER2+	II	W	0	CBR	KIARA	NCT03203616
Pyrotinib(EGFRi), capecitabine (CT)	HER2+	II	R	362	ORR	-	NCT05042791
Anti-HER2/HER3 Dendritic Cell Vaccine, Pembrolizumab (ICI)	TNBC	II	R	23	ORR	-	NCT04348747
68GaNOTA-Anti-HER2 VHH1 (radioconjugate)	BCBM	II	R	30	TTP	-	NCT03331601
Pyrotinib(EGFRi), Trastuzumab (MA), Abraxane (CT)	BCBM	II	U	100	ORR, PFS	-	NCT04639271
Pertuzumab (MA) Trastuzumab (MA)	HER2+	I	T	1	AE	-	NCT02598427
Lapatinib (TKI)	BCBM	II	C	82	ORR	-	NCT01218529
Palbociclib (CDK4/6i)	HER2+	II	T	12	ORR	-	NCT02774681
Tucatinib (TKI), Pembrolizumab (ICI), Trastuzumab (MA)	HER2+	I, II	W	0	ORR	TOPAZ	NCT04512261
UDT1 (microtubule stabilizing agent) combined with capecitabine (CT)	HER2-	I, II	R	30	ORR	-	NCT05535413
Cabazitaxel (CT)	HER2-	II	W	0	ORR	CRANIAL	NCT01913067
Pyrotinib(EGFRi)	HER2+	II	NR	60	TTAP	-	NCT05255523
Lapatinib (KI), Temozolomide (CT)	HER2+	I	C	18	MTD	LAPEM	NCT00614978
Berubicin hydrochloride (RTA 744)	BMBC	II	T	14	LTCR	-	NCT00538343

Trastuzumab (AC)	HER2+	II	T	3	ORR	-	NCT01363986
Lapatinib (KI)	BMBC	II	C	37	ORR	-	NCT00098605
Sacituzumab Govitecan (ADC)	HER2-	II	R	44	ORR	-	NCT04647916
Veliparib (PARPi), cisplatin (CT)	TNBC, BRCA Mutation-Associated	II	A	333	PFS		NCT02595905
BKM 120 (PI3Ki), Trastuzumab (MA)	ER+/HER2-, HER2+, TNBC	II	C	10	CBR	STAR Cape+BKM120	NCT02000882
Epothilone B (microtubule-stabilizing agent)	BCBM	II	C	55	PFS	-	NCT00450866
Afatinib (TKI)	HER2+	II	C	121	CBR	Lux-Breast 3	NCT01441596
Trastuzumab (MA), Trastuzumab Deruxtecan (ADC)	HER2+	IV	R	10	ReR	-	NCT05376878
Capecitabine (CT), topotecan (CT), Lapatinib (KI)	HER2+	II	T	22	ORR	-	NCT00437073
ARRY-380 (EGFRi), Trastuzumab (MA)	HER2+	I	A	41	MTD	-	NCT01921335
Dendritic vaccine, allogeneic hematopoietic stem cells, cytotoxic lymphocytes	BCBM	II, III	U	60	ACM	-	NCT01782274
ANG1005 (CT)	HER2-	III	NR	150	OS	ANGLeD	NCT03613181
GRN1005 (PDC)	BCBM	II	W	0	CBR	-	NCT01679743
Lapatinib (KI)	HER2+	II	C	242	ORR	-	NCT00263588
Abemaciclib (CDK4/6i), Elacestrant (ERA)	HR+ HER2-	I, II	R	106	DLT	ELECTRA	NCT05386108
Temozolomide (CT)	BCBM	II	T	6	Metastasis recurrence	STOP	NCT00638963

Abemaciclib (CDK4/6i)	HR+ HER2-, HR+ HER2+	II	C	162	ORR	-	NCT02308020
Pegylated Irinotecan (TIi)	BCBM	II	C	27	DCR	-	NCT02312622
Capecitabine (CT)	HER2+	IV	U	43	TTP	-	NCT04767828
Tucatinib (TKi), Pertuzumab (MA), Trastuzumab (MA)	HER2+	II	R	55	PFS	InTTercePT	NCT05041842
BKM120 (PI3Ki), Trastuzumab (MA)	HER2+	I, II	T	72	DLT, ORR	-	NCT01132664
Trastuzumab deruxtecan (ADC)	HER2+	II	C	41	PFS, ORR	DEBBRAH	NCT04420598
Pertuzumab (MA), Trastuzumab(MA)	HER2+	II	C	40	ORR	-	NCT02536339
Bevacizumab (MA), Etoposide (CT), Cisplatin (CT)	BCBM	II	C	40	ORR	-	NCT01281696
KD019 (EGFRi), Trastuzumab (MA)	HER2+	I, II	T	17	S&T	-	NCT02154529
DCVax-Direct (Dendritic cell therapy)	BCBG	I	U	24	S&T	-	NCT03638765
Lapatinib (KI), Capecitabine (CT)	HER2+	I	C	11	MTD	-	NCT02650752
Cabazitaxel (CT)	BCBM	II	T	8	ORR	CaBaMet	NCT02166658
Capecitabine (CT)	BCBM	II	C	3	PFS	-	NCT01077726
Lapatinib (KI), Trastuzumab (MA), Capecitabine (CT)	HER2+	III	C	540	Nubmer of relapsed patients		NCT00820222
Sunitinib (TKI), Capecitabine (CT)	BCBM	II	T	12	PFS	-	NCT00570908
Tremelimumab (MA)	HER2+	N/A	C	28	PFS	-	NCT02563925

Afatinib (TKI)	BCBM	I, II	U	70	Ratio of afatinib concentration	CamBMT1	NCT02768337
Temozolomide (CT)	BCBM	II	C	162	ORR	-	NCT00831545
Durvalumab (ICI)	BCBM	II	T	4	ORR	-	NCT02669914
Trastuzumab (MA)	HER2+	I, II	C	84	S&T, MTD	-	NCT01386580
Tucatinib (TKI)	HER2+	II	C	612	PFS	HER2CLIMB	NCT02614794
Trastuzumab (MA), intra-arterial cerebral Infusion of	HER2+	I	T	2	MTD, DLT	-	NCT02571530
Lapatinib (KI), Trastuzumab (MA)	HER2+	III	C	8382	DFS	ALTTO	NCT00490139
SCR-6852 (SERD), Palbociclib (CDK4/6i)	HR+ HER2-	I	R	146	MTD, DLT	-	NCT05293964
Atezolizumab (MA), Trastuzumab (MA), Vinorelbine (CT)	HER2+	II	R	110	ORR	ATREZZO	NCT04759248
Trastuzumab deruxtecan (ADC), Durvalumab (ICI), Paclitaxel (CT), Pertuzumab (MA), Tucatinib (TKI)	HER2+	I, II	R	245	AE	DB-07	NCT04538742
ZN-A-1041 (TKI)	HER2+	I	R	210	AE	-	NCT05593094
Lapatinib (KI), Letrozole (AI)	HER2+	III	C	1286	PFS	-	NCT00073528
Atezolizumab (MA)	HER2+	III	A	600	PFS	-	NCT03199885
Lapatinib (KI), doxorubicinhydrochloride (CT)	BCBM	II	T	24	ORR	-	NCT00903656
Tucatinib (TKI), Abemaciclib (CDK4/6i), Trastuzumab (MA), AI	HER2+	I	W	0	MTD	-	NCT03846583
Trastuzumab (MA), irinotecan hydrochloride (TLi)	HER2+	II	C	9	ORR	-	NCT00303992

Capecitabine (CT), camrelizumab (ICI), apatinib (VEGFi)	TNBC	II, III	R	260	DFS	Artemis	NCT04803539
Bintrafusp Alfa (bifunctional fusion protein, Pimasertib(MEK1/2)	HR+, TNBC	I, II	A	10	CBR	-	NCT04789668
Tucatinib (TKI), Trastuzumab (MA), Vinorelbine (CT)	HER2+	II	R	49	ORR	TrasTUCAN	NCT05583110
ONC201 (Akt/ERK inhibitor)	TNBC	II	T	4	ORR	-	NCT03733119
Tucatinib (TKI), Trastuzumab emtansine (ADC)	HER2+	III	R	1031	DFS	CompassHER2	NCT04457596
TPI 287 (abeotaxane)	BCBM	II	C	24	ORR	-	NCT01332630
NUV-422 (CDK2/4/6i)	BCBM	I, II	T	72	AE	-	NCT04541225
Tislelizumab (MA), Capecitabine (CT)	TNBC	II	R	460	DFS	Apollo	NCT04501523
Epirubicin (CT), nab-Paclitaxel (CT), Cyclophosphamide 8CT9, Docetaxel (CT)	High-risk early breast cancer	III	C	2886	DFS	GAIN-2	NCT01690702
HX008 (MA), Niraparib (PARPi), Trastuzumab (MA), Pyrrolitinib (PARPi)	HER2+, HER2-	II	R	37	ORR	CHANGEABLE	NCT04508803
ZN-A-1041	HER2+	I	R	84	S&T	-	NCT04487236
Methotrexate (CT),liposomal cytarabine	BCBM	II	C	3	PFS	-	NCT00992602
Trastuzumab Emtansine (ADC), Atezolizumab (MA)	HER2+	III	A	96	PFS, OS	KATE3	NCT04740918
Tucatinib (TKI), Trastuzumab deruxtecan (ADC)	HER2+	III	R	565	PFS	-	NCT03975647
Trastuzumab Deruxtecan (ADC), Trastuzumab Emtansine(ADC)	HER2+	III	R	1600	DFS	DESTINY-Breast05	NCT04622319
Irinotecan (Tli)	BCBM	I	NR	136	AE	-	NCT04728035

Indinavir sulfate, ritonavir (antiviral)	BCBM	II	U	60	OS	-	NCT00637637
Trastuzumab (MA)	HER2+	III	T	13	DFS	TSARINE	NCT01613482
HER2 BATs (bi-specific antibody armed activated T-cells)	BCBM	I	T	3	AE	-	NCT03661424
QBS10072S (CT)	BCBM	I	C	15	MTD	-	NCT04430842
Tucatinib (TKI), Pembrolizumab (ICI), Trastuzumab (MA), Capecitabine (CT)	HER2+	II	R	50	ORR	TUGETHER	NCT04789096
4-Demethyl-4-cholestryloxycarbonylpenclomedine	BCBM	II	C	29	OS, tumor diameter	DM-CHOC-PEN	NCT02038218
Cabazitaxel (CT), Lapatinib (TKI)	HER2+	II	T	11	ORR	-	NCT01934894
Atezolizumab (MA), Trastuzumab (MA)	HER2+	II	A	33	ORR	-	NCT03417544
Lapatinib ditosylate (EGFRi), everolimus (CT), capecitabine (CT)	HER2+	I, II	C	9	ORR	-	NCT01783756
DZD1516 (TKI), Trastuzumab (MA), Capecitabine (CT), Trastuzumab Deruxtecan (ADC)	HER2+	I	A	23	AE, DLT, MTD		NCT04509596
Methotrexate (CT)	BCBM	II	R	16	OS	-	NCT02422641
Eribulin mesylate (MTA)	HER2-	II	C	56	ORR	-	NCT01268150
2B3-101 (Glutathione pegylated liposomal doxorubicin)	BCBM	II	U	6	S&T	-	NCT01818713
Trastuzumab (MA), R115777 (tipifarnib, farnesyltransferase inhibitor)	HER2+	I	C	24	-	-	NCT00005842
ELVN-002 (EGFRi)	HER2+	I	R	178	DLT, AE	-	NCT05650879

Chimeric Antigen Receptor T-Cell Therapy	HER2+	I	R	39	Safety and recommended phase 2 dosing determination	-	NCT03696030
Fulvestrant (ERA), Arimidex (AI)	HR+	III	A	462	PFS	FALCON	NCT01602380
Fulvestrant (ERA), Capivasertib (KI)	HR+ HER2-	III	A	818	PFS	CAPItello-291	NCT04305496
AZD5363 (KI), Fulvestrant (ERA)	HR+	I, II	A	149	MTD, PFS	FAKTION	NCT01992952
Talazoparib (PARPi)	BCBM	II	T	84	ORR	ABRAZO	NCT02034916
Talazoparib (PARPi)	BCBM	III	C	431	PFS	EMBRACA	NCT01945775
Pertuzumab (ICI), Trastuzumab (MI), Docetaxel (CT)	HER2+	III	C	808	PFS	CLEOPATRA	NCT00567190
Trastuzumab Emtansine(ADC)	HER2+	III	C	602	PFS, OS	TH3RESA	NCT01419197
Pertuzumab (ICI), Trastuzumab (MI), Docetaxel (CT)	HER2+	III	C	2185	AEPIs	KAMILLA	NCT01702571

Supplementary Table S1. Breast cancer brain metastasis targeting agents and corresponding clinical trials. Abbreviations: HR+, hormone recep-tor-positive; HER2-, HER2-negative breast cancer; HER2+, HER2-positive breast cancer; TNBC, triple-negative breast cancer; BCBM, breast cancer with brain metastasis (molecular type not specified); ADC, antibody-drug conjugate; AI, aromatase inhibi-tor; CDK4/6i, cyclin-dependent kinases CDK4 and CDK6 inhibitor; CT, chemotherapy; EGFRi, pan-HER receptor tyrosine kinase inhibitor; ERA, estrogen receptor antagonist; ICI, immune checkpoint inhibitor; KI, kinase inhibitor; MA, monoclonal antibody; MEK1/2, mitogen-activated protein kinases 1 and 2 inhibitor; MTA, microtubule targeting agent; PARPi, poly ADP ribose poly-merase inhibitor; PDC, peptide-drug conjugate; PI3Ki, PI3-kinase inhibitor; TIi, topoisomerase I inhibitor; SERD, selective es-trogen receptor degrader; TKI, tyrosine kinase inhibitor; VEGFi, vascular endothelial growth factor A inhibitor; A, active; C, completed; N/A, non applicable; NR, not yet recruiting; R, recruiting; T, terminated; U, Unknown; W, withdrawn; ACM, all-cause mortality; AEs, adverse events; AEPIs, Adverse Events of Primary Interest; CBR, Clinical Benefit Rate; DCR, Disease Control Rate; DFS, disease free survival; DLT, dose limiting toxicity; MTD, maximum tolerated dose; LTCR, local tumor control rate; ORR, objective response rate; OS, overall survival; PFS, progression-free survival; ReR, response rate; S&T, safety and tolerability; TTP, time to progression; TTAP, tumor targeting potential. Data has been obtained from clinicaltrials.gov and clinicaltrialsregister.eu (accessed on 1st May 1, 2023).

Intervention (drug)	Breast Cancer type	Phase	Patients	Primary Outcome	Acronym	Trial Number
Trastuzumab (MA) and GRN1005 (PDC)	HER2+	II	85	ORR	GRABM-B	NCT01480583
ANG1005 (PDC)	BCBM	II	72	ORR	-	NCT02048059
Etirinotecan Pegol (polymer conjugate of TIi)	BCBM	III	178	OS	ATTAIN	NCT02915744
Eribulin Mesylate (MTA)	BCBM	II	9	PFS	-	NCT02581839
Trastuzumab Emtansine (ADC)	HER2+	I	36	optimal sequences of combined treatment	BIRTH	NCT02135159
Sorafenib (KI)	BCBM	I	21	MTD	-	NCT01724606
Lapatinib (TKI)	HER2+	I	35	MTD	-	NCT00470847
Capecitabine (CT), lapatinib (TKI)	HER2+	II	45	ORR	-	NCT00967031
Everolimus (KI), Vinorelbine (CT), Trastuzumab (MA)	HER2+	II	32	ReR	-	NCT01305941
Bevacizumab (MA)	HER2+	II	38	ORR	-	NCT01004172
Cabozantinib (TKI)	HER2+/ ER+/ TNBC	II	36	ORR	-	NCT02260531
INIPARIB (PARPi)	TNBC	II	44	TTP		NCT01173497
Efaproxiral (allosteric hemoglobin modifier)	BCBM	III	368	OS	ENRICH	NCT00083304
Lapatinib (TKI)	BCBM	II	82	ORR	-	NCT01218529
Lapatinib (KI), Temozolomide (CT)	HER2+	I	18	MTD	LAPEM	NCT00614978

Lapatinib (KI)	BMBC	II	37	ORR	-	NCT00098605
BKM 120 (PI3Ki), Trastuzumab (MA)	ER+/HER2-, HER2+, TNBC	II	10	CBR	STAR Cape+BKM120	NCT02000882
Epothilone B (microtubule-stabilizing agent)	BCBM	II	55	PFS	-	NCT00450866
Afatinib (TKI)	HER2+	II	121	CBR	Lux-Breast 3	NCT01441596
Lapatinib (KI)	HER2+	II	242	ORR	-	NCT00263588
Abemaciclib (CDK4/6i)	HR+ HER2-, HR+ HER2+	II	162	ORR	-	NCT02308020
Pegylated Irinotecan (TIi)	BCBM	II	27	DCR	-	NCT02312622
Trastuzumab deruxtecan (ADC)	HER2+	II	41	PFS, ORR	DEBBRAH	NCT04420598
Pertuzumab (MA), Trastuzumab(MA)	HER2+	II	40	ORR	-	NCT02536339
Bevacizumab (MA), Etoposide (CT), Cisplatin (CT)	BCBM	II	40	ORR	-	NCT01281696
Lapatinib (KI), Capecitabine (CT)	HER2+	I	11	MTD	-	NCT02650752
Capecitabine (CT)	BCBM	II	3	PFS	-	NCT01077726
Lapatinib (KI), Trastuzumab (MA), Capecitabine (CT)	HER2+	III	540	Nubmer of relapsed patients		NCT00820222
Tremelimumab (MA)	HER2+	N/A	28	PFS	-	NCT02563925
Temozolomide (CT)	BCBM	II	162	ORR	-	NCT00831545
Trastuzumab (MA)	HER2+	I, II	84	S&T, MTD	-	NCT01386580
Tucatinib (TKI)	HER2+	II	612	PFS	HER2CLIMB	NCT02614794

Lapatinib (KI), Trastuzumab (MA)	HER2+	III	8382	DFS	ALTTO	NCT00490139
Lapatinib (KI), Letrozole (AI)	HER2+	III	1286	PFS	-	NCT00073528
Trastuzumab (MA), irinotecan hydrochloride (TLi)	HER2+	II	9	ORR	-	NCT00303992
TPI 287 (abeotaxane)	BCBM	II	24	ORR	-	NCT01332630
Epirubicin (CT), nab-Paclitaxel (CT), Cyclophosphamide 8CT9, Docetaxel (CT)	High-risk early breast cancer	III	2886	DFS	GAIN-2	NCT01690702
Methotrexate (CT),liposomal cytarabine	BCBM	II	3	PFS	-	NCT00992602
QBS10072S (CT)	BCBM	I	15	MTD	-	NCT04430842
4-Demethyl-4-cholestryloxycarbonylpenclomedine	BCBM	II	29	OS, tumor diameter	DM-CHOC-PEN	NCT02038218
Lapatinib ditosylate (EGFRi), everolimus (CT), capecitabine (CT)	HER2+	I, II	9	ORR	-	NCT01783756
Eribulin mesylate (MTA)	HER2-	II	56	ORR	-	NCT01268150
Trastuzumab (MA), R115777 (tipifarnib, farnesyltransferase inhibitor)	HER2+	I	24	-	-	NCT00005842
Talazoparib (PARPi)	BCBM	III	431	PFS	EMBRACA	NCT01945775
Pertuzumab (ICI), Trastuzumab (MI), Docetaxel (CT)	HER2+	III	808	PFS	CLEOPATRA	NCT00567190
Trastuzumab Emtansine(ADC) Pertuzumab (ICI), Trastuzumab (MI), Docetaxel (CT)	HER2+	III	602	PFS, OS	TH3RESA	NCT01419197

Trastuzumab (MA), R115777 (tipifarnib, farnesyltransferase inhibitor)	HER2+	III	2185	AEPIs	KAMILLA	NCT01702571

Supplementary Table S2. Breast cancer brain metastasis targeting agents and corresponding complete clinical trials. Abbreviations: HR+, hormone recep-tor-positive; HER2-, HER2-negative breast cancer; HER2+, HER2-positive breast cancer; TNBC, triple-negative breast cancer; BCBM, breast cancer with brain metastasis (molecular type not specified); ADC, antibody-drug conjugate; AI, aromatase inhibi-tor; CDK4/6i, cyclin-dependent kinases CDK4 and CDK6 inhibitor; CT, chemotherapy; EGFRi, pan-HER receptor tyrosine kinase inhibitor; ERA, estrogen receptor antagonist; ICI, immune checkpoint inhibitor; KI, kinase inhibitor; MA, monoclonal antibody; MEK1/2, mitogen-activated protein kinases 1 and 2 inhibitor; MTA, microtubule targeting agent; PARPi, poly ADP ribose poly-merase inhibitor; PDC, peptide-drug conjugate; PI3Ki, PI3-kinase inhibitor; TIi, topoisomerase I inhibitor; SERD, selective es-trogen receptor degrader; TKI, tyrosine kinase inhibitor; VEGFi, vascular endothelial growth factor A inhibitor; ACM, all-cause mortality; AEs, adverse events; AEPIs, Adverse Events of Primary Interest; CBR, Clinical Benefit Rate; DCR, Disease Control Rate; DFS, disease free survival; DLT, dose limiting toxicity; MTD, maximum tolerated dose; LTCR, local tumor control rate; ORR, ob-jective response rate; OS, overall survival; PFS, progression-free survival; ReR, response rate; S&T, safety and tolerability; TTP, time to progression; TTAP, tumor targeting potential. Data has been obtained from clinicaltrials.gov and clinicaltrialsregister.eu (accessed on 1st May 1, 2023).
